# Fusion Surgery Required for Recurrent Pediatric Atlantoaxial Rotatory Fixation after Failure of Temporary Fixation with Instrumentation

**DOI:** 10.1155/2017/1017307

**Published:** 2017-12-26

**Authors:** Yoshiyuki Matsuyama, Tetsuhiro Ishikawa, Ei Ozone, Masaaki Aramomi, Seiji Ohtori

**Affiliations:** ^1^Orthopaedic Surgery, Sanmu Medical Center, 167 Naruto, Sanmu, Chiba 289-1326, Japan; ^2^Department of Orthopaedic Surgery, Graduate School of Medicine, Chiba University, 1-8-1 Inohana, Chuo-ku, Chiba 260-8670, Japan

## Abstract

In cases of chronic irreducible and recurrent unstable atlantoaxial rotatory fixation (AARF), closed reduction and its maintenance are often unsuccessful, requiring surgical treatment. The purpose of the present report is to describe a rare case of pediatric AARF that required multiple treatments. A 6-year-old boy was diagnosed as having type 2 AARF. After conservative treatment, the patient was treated with temporary fixation surgery (C1-C2 Magerl) without a bone graft in consideration of motion preservation after screw removal. AARF recurred after the screw removal and required fusion surgery (Magerl–Brooks) with an iliac bone graft. Ultimately, bone union was achieved and the screws were removed 11 months after the surgery. We recommend surgeons be cautious when choosing temporary fixation surgery for AARF in small children. Further investigation is needed to determine the optimal time before screw removal.

## 1. Introduction

In 1977, Fielding and Hawkins classified atlantoaxial rotatory fixation (AARF) into four types depending on the degree of anterior or posterior displacement of the atlas [1]. AARF causes torticollis and neck pain because of dislocation or subluxation of the atlantoaxial joint. It has been found mainly in children and may accompany mild trauma, upper respiratory infection, a damaged joint capsule and ligament, or hypermyotonia [2–4]. Anatomical features of the C1-C2 facet in children are less stable than those in adults because of a loose joint capsule, a wide joint range of motion, and a horizontal articular surface [1, 5, 6]. AARF sometimes results from dysfunction of the transverse ligament of the atlas, which causes inflammation of the tonsils, the pharynx, and the upper airway [3, 7].

Most acute AARFs can be treated successfully by conservative therapy including medication, closed manipulation, or cervical traction followed by bracing [2, 4]. In chronic cases, closed reduction and its maintenance are often unsuccessful, requiring surgical treatment for such patients with chronic irreducible and recurrent unstable AARF [2, 8, 9].

In this paper, we review the literature and report a case of AARF in a 6-year-old boy requiring fusion surgery after failed treatment with temporary fixation and motion preservation surgery.

## 2. Case Report

**Figure 1:**
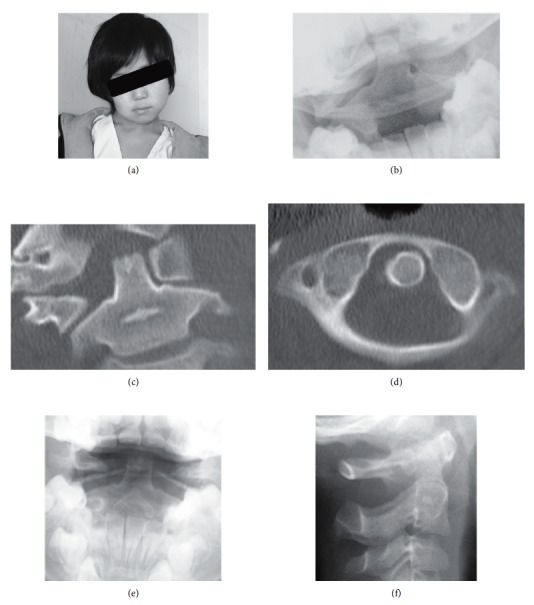
Patient showing torticollis before treatment, with head tilting, neck rotation, and a characteristic “cock-robin” position (a). Simple cervical open mouth view radiograph showed lateral tilting of the cervical spine and rotated atlas on the axis (b). Coronal (c), axial (d), and 3D (e). CT images showed lateral tilting of C1 and anterior rotated atlas on the axis. Plain radiograph after closed reduction under general anesthesia (e, f).

A 6-year-old boy visited our department with torticollis and neck pain that occurred after a small fight with his brother 8 days earlier. The patient had a previous history of a chronic sinus problem with a nasal discharge. The white blood cell (WBC) count was 12,800, and the C-reactive protein (CRP) level was 0.5. His physical examination showed torticollis, with head tilting, neck rotation, and a characteristic “cock-robin” position (Figure 1). We diagnosed AARF (Fielding type 2) by plain radiography (Figure 1) and computed tomography (CT) (Figures 1 and 1). 3D CT showed anterior subluxation of the right C1-C2 facet (Figures 1 and 1), and a C2 facet deformity had not appeared. His extremities appeared normal on neurological examination.

Three weeks after conservative treatment with a neck collar, AARF had not improved. We recommended inhospital care and cervical traction, but the parents denied long-term admission because they have other small children and were busy with work. Therefore, closed reduction under general anesthesia was performed and AARF improved (Figures 1 and 1). However, AARF recurred when he woke up and walked to the bathroom 3 hours after the closed reduction.

**Figure 2:**
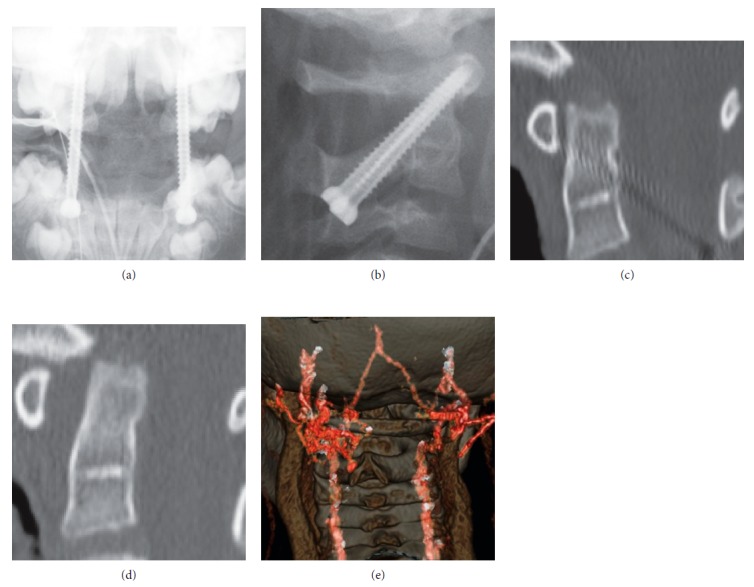
Result of C1-C2 Magerl surgery without a bone graft (a, b). Odontoid process shows osteolysis after C1-C2 Magerl surgery (c). Type 2 AARF recurred 2 weeks after the screw removal. The reconstruction CT shows osteolysis of the odontoid process (d). Preoperative 3D CT showed hypervascularity posterior to C1 (e).

We performed C1-C2 Magerl surgery without bone fusion as a temporary fixation (Figures 2 and 2) because the patient was 6 years old and we expected C1-C2 motion preservation after the removal of the screws [10]. We chose the Magerl technique instead of C1 lateral mass screw-C2 pedicle screw and rod fixation because there was abnormal hypervascularity posterior to C1 on preoperative 3D CT angiogram (Figure 2). A plain radiograph and CT image after the surgery showed improvement of AARF, but the odontoid process appeared osteolytic (Figure 2).

Considering bone union between C1 and C2, we removed the screws at 17 weeks after the surgery. However, the type 2 AARF recurred 2 weeks after the screw removal. Reconstructed CT images showed osteolysis of the odontoid process (Figure 2). Just before the removal surgery, the WBC count was 10,800 and the CRP level was 0.0; inflammation of the transverse ligament and the influence of upper respiratory infection were suspected. Although we did not notice initially, retrospectively, osteolysis of the odontoid process was shown in the preoperative CT scan (Figure 1).

**Figure 3:**
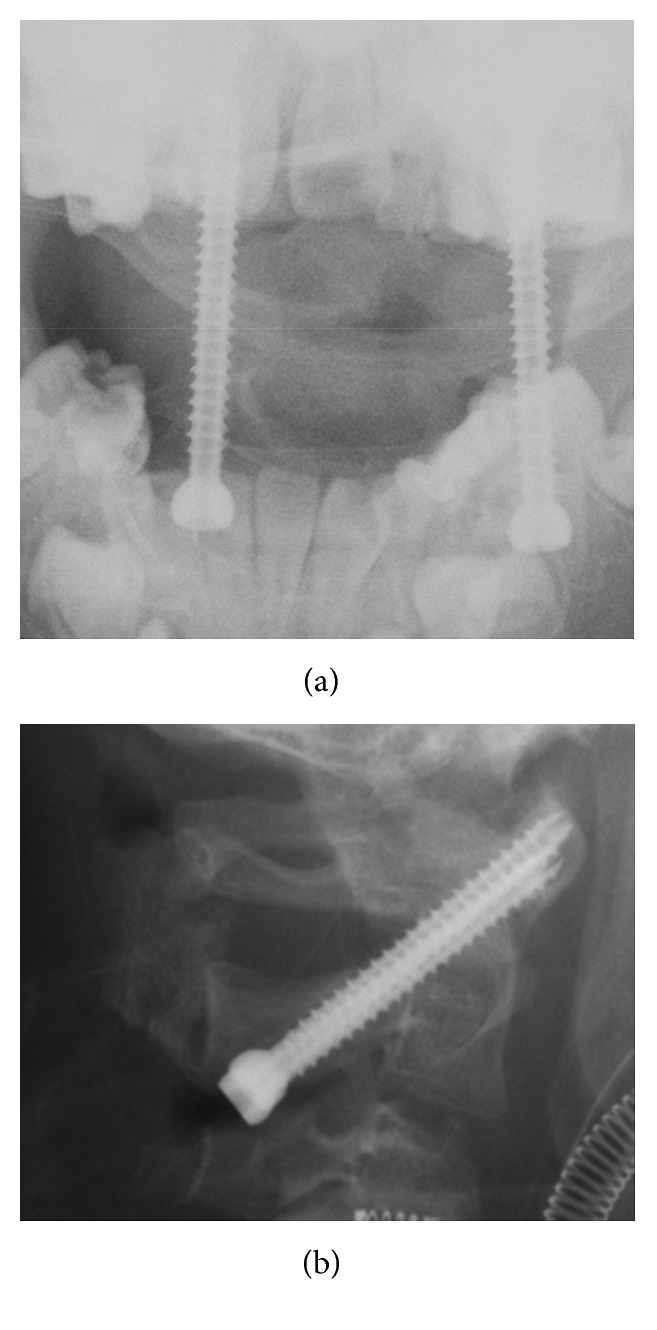
Result of posterior fixation surgery using the Magerl–Brooks method with an iliac bone graft.

We performed posterior fixation surgery using the Magerl–Brooks method with an iliac bone graft 5 weeks after the recurrence (Figure 3) [10, 11]. Ultimately, bone union was achieved 11 months after the surgery, and the screws were removed because we were concerned about growth disorder of the cervical bone without removal. On the most recent CT images, the odontoid process showed sclerosis and there were no signs of inflammation of the transverse ligament.

## 3. Discussion

AARF causes torticollis and neck pain because of dislocation or subluxation of the atlantoaxial joint [2, 11, 12]. Fielding and Hawkins proposed four types of AARF. Type 1: unilateral facet subluxation with an intact transverse ligament. This is the most common type; the dens acts as a pivot. Type 2: unilateral facet subluxation with an atlantodental interval (ADI) of 3–5 mm. This type is associated with the transverse ligament injury; the facet acts as a pivot. Type 3: bilateral anterior facet displacement of >5 mm. This type is rare, with a risk of neurologic deficit. Type 4: posterior displacement of the atlas, associated with dens deficiency [4]. This type is rare, with a risk of neurological deficit. Most acute AARFs can be treated successfully by conservative therapy including closed manipulation or cervical halter traction followed by a cervical orthosis [6, 7]. The pathophysiology of the chronic and recurrent AARF remains unclear despite many previous studies of AARF [2–4,8,9]. Ishii et al. reported that nontraumatic AARF is associated with pharyngeal infection [7]. In the present case, we considered that AARF was a result of trauma; however, the patient had a chronic sinus problem with nasal discharge, so laxation of the transverse ligament and the odontoid destruction caused by chronic inflammation might have influenced the delay and recurrence of AARF.

With early diagnosis and reduction, most patients can be successfully treated and cured, but a delay in diagnosis correlates with recurrence and leads to chronic AARF [13]. When conservative treatment fails, open reduction and posterior fixation are necessary. Tauchi et al. successfully treated chronic AARF in children with posterior fusion, such as C1-C2 transarticular fixation, and C1 lateral mass screw and C2 pedicle screw fixation [13]. Atlantoaxial arthrodesis is associated with several problems such as pseudarthrosis, long operation time, and loss of range of motion (ROM) at the atlantoaxial joint. Han et al. reported a case series in 13 patients with type 2 odontoid fractures, using temporary pedicle screw fixation without bone fusion for motion preservation [14]. Ni et al. also reported posterior reduction and temporary fixation with odontoid fracture in 22 consecutive patients, and fracture healing was obtained in 21 [15]. Furthermore, after removing the instrumentation, the ROM of C1-C2 in rotation was obtained, and the neck pain and stiffness were relieved [15]. In reference to these methods, to avoid the loss of C1-C2 motion in small children, we chose temporary fixation surgery and removed the screws before C1-C2 had fused at 17 weeks after the surgery. Our findings indicate that 17 weeks is too early to obtain stability. However, we could not find any reports regarding an appropriate time for temporary fixation for small children. We recommend caution when choosing temporary fixation surgery. Further investigation is needed to clarify the appropriate time necessary to achieve stable reduction of AARF in small children.

In summary, we experienced multiple recurrences of AARF in a small child. Temporary fixation and motion preservation did not accomplish stability, but ultimately we could treat the condition with posterior fixation surgery using the Magerl–Brooks method.
